# Mutations in the NOG gene are commonly found in congenital stapes ankylosis with symphalangism, but not in otosclerosis

**DOI:** 10.1111/j.1399-0004.2011.01831.x

**Published:** 2012-01-30

**Authors:** S Usami, S Abe, S Nishio, Y Sakurai, H Kojima, T Tono, N Suzuki

**Affiliations:** a Department of Otorhinolaryngology, Shinshu University School of MedicineMatsumoto, Japan; b Department of Otorhinolaryngology, Abe ENT ClinicOta-ku, Japan; c Department of Otorhinolaryngology, Jikei University School of MedicineMinato-ku, Tokyo, Japan; d Department of Otorhinolaryngology, University of Miyazaki Faculty of MedicineMiyazaki, Japan

**Keywords:** otosclerosis, stapes ankylosis, stapes ankylosis with broad, SYM1, symphalangism, Teunissen and Cremer syndrome, thumb and toes

## Abstract

Human noggin (*NOG*) is a responsible gene for multiple synostosis syndrome (SYNS1) and proximal symphalangism (SYM1), two conditions that are recently known to be within a wider range of clinical manifestations of stapes ankylosis with symphalangism. This study was performed to determine the range of phenotype caused by *NOG* mutations, using Japanese patients with various phenotypes including sporadic inherited SYM1, dominantly inherited SYM1, stapes ankylosis with broad thumb and toes (Teunissen and Cremer syndrome). In addition, 33 patients with typical otosclerosis (without symphalangism) were studied. Direct sequencing analysis disclosed three novel mutations of the *NOG* gene in three SYM1 families. None of the otosclerosis patients without symphalangism had *NOG* mutations, indicating that *NOG* mutations may be restrictively found within patients with various skeletal abnormalities. These results together with the literature review indicated that there are no clear genotype–phenotype correlations for NOG mutations. With regard to surgical outcome, most of the patients in these three families with *NOG* mutations showed remarkable air–bone gap recovery after stapes surgery. Molecular genetic testing is useful to differentiate syndromic stapes ankylosis from otosclerosis, and even mild skeletal anomalies can be a diagnostic indicator of NOG-associated disease.

## Introduction

Human noggin (*NOG*) is a responsible gene for a wide range of clinical manifestations of stapes ankylosis with symphalangism. Proximal symphalangism (SYM1: MIM #185800) (1) is known as an autosomal dominant disorder with high penetrance. The most common clinical features are the immobility of the proximal interphalangia (PIP) joints of the hands and toes, and congenital conductive hearing loss due to stapes ankylosis. Multiple synostosis syndrome (SYNS1: MIM#186500) (1) is characterized by a more severe phenotype of the proximal symphalangism, such as progressive and expanded bony fusion of joints and unique facial manifestations. In addition, mutations in NOG have been identified in Tarsal–Carpal Coalition syndrome (TCC: MIM#186570) (2), stapes ankylosis with broad thumb and toes (MIM#184460) (Teunissen and Cremer syndrome) (3), and Brachydactyly type B2 (BDB2: MIM#611377) (4).

Otosclerosis (MIM#166800) is known as the single most common cause of progressive conductive hearing loss, characterized by abnormal bone remodeling in the otic capsule. Although there are a small number of familial cases that are likely to be monogenic, the majority of cases are sporadic. A series of studies has suggested that this condition involves both genetic and environmental factors (5).

Typical otosclerosis was included in this study because it is an interesting question as to whether some of the typical otosclerosis is a continuum of a category of disease caused by *NOG* mutations. We thought this may be true because (i) within SYM1, *NOG* mutations were found in patients with minor skeletal anomalies without symphalangism (3), and (ii) stapes ankylosis is an important phenotype of the animal model for NOG^+/−^ mice (6).

To date, no detailed survey was available for *NOG* mutations in the stapes ankylosis patients with symphalangism in Asian populations. Therefore this study was undertaken to address whether *NOG* mutations are also causative and commonly found in those populations and if so, whether there is a different mutation spectrum.

In addition, previously reported *NOG* mutations were reviewed to determine their spectrum as well as whether there are any particular genotype–phenotype correlations caused by *NOG* mutations.

## Materials and methods

### Subjects

We ascertained three Japanese families to be associated with conductive hearing loss and symphalangism, including an autosomal dominant SYM1 family, a sporadic SYM1 case with normal parents, and an autosomal dominant stapes ankylosis with broad thumb and toes (Teunissen and Cremer syndrome) family. Thirty-three Japanese otosclerosis patients, who underwent stapes surgery, were also screened for mutations in the *NOG* gene. Their clinical symptoms, including ages at surgery (36–77 years old: average 54.4 years old), onset age (15–57 years old: average 37.3 years old), gender (10 male and 23 female), laterality (9 unilateral and 24 bilateral), and hearing threshold (average 63.1 dB), are summarized in Table 1. Average onset age, was hearing threshold, was evaluated using pure-tone audiometry classified by a pure-tone average over 250, 500, 1000, 2000, and 4000 Hz. By detailed anamnestic and medical examination, no patients had any associated skeletal abnormalities. All of the patients were sporadic cases and no similar condition was observed within their familial members. Satisfactory outcomes after stapes surgery were obtained in all 33 subjects.

**Table 1:** Clinical symptoms of Otosclerosis patients

Patient number	Age	Gender	Onset age	Affected side	Hearing threshold (right)	Hearing threshold (left)
1	48	M	36	Bilateral	68.3	56.3
2	59	F	45	Bilateral	56.3	56.3
3	43	F	38	Left	10.8	64.0
4	36	M	25	Bilateral	61.3	66.3
5	46	F	40	Right	38.0	19.0
6	44	F	33	Bilateral	59.0	66.0
7	65	F	49	Bilateral	81.0	63.0
8	77	F	57	Bilateral	104.0	105.0
9	45	F	41	Bilateral	56.0	69.0
10	44	F	30	Left	30.0	80.0
11	61	F	44	Bilateral	23.0	68.0
12	58	F	49	Right	76.0	35.0
13	43	M	25	Left	14.0	49.0
14	58	F	47	Bilateral	53.0	50.0
15	54	F	38	Bilateral	57.0	45.0
16	53	F	40	Right	58.0	6.0
17	44	F	25	Bilateral	53.0	56.0
18	62	F	48	Bilateral	42.5	52.5
19	43	F	33	Bilateral	27.0	45.0
20	57	F	40	Left	26.0	65.0
21	65	F	15	Bilateral	53.0	50.0
22	54	M	46	Bilateral	50.0	30.0
23	48	F	23	Bilateral	71.0	56.0
24	62	F	39	Bilateral	38.0	37.0
25	76	F	43	Bilateral	78.0	75.0
26	71	M	41	Bilateral	91.3	97.5
27	71	F	40	Bilateral	126.3	110.0
28	45	M	45	Left	42.5	73.75
29	41	F	30	Bilateral	98.8	95
30	44	M	30	Left	30.0	76.3
31	44	M	30	Right	58.8	38.8
32	64	M	35	Bilateral	51.0	46.0
33	70	M	30	Bilateral	48.8	53.8

F, female; M, male.

We obtained informed consent for participation in this project from each subject and also from 192 normal control subjects who were unrelated Japanese individuals without any noticeable hearing loss evaluated by auditory testing. Otologic examination, audiometric analysis, and radiologic imaging were carried out for each patient.

### Family 1

**Figure 1:**
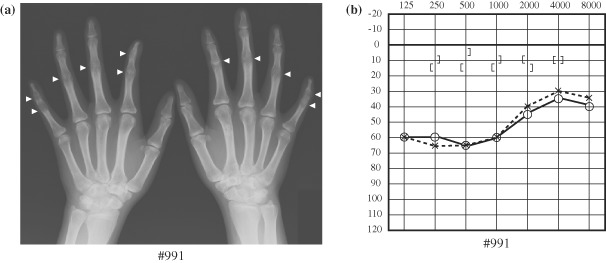
(**a**) Photograph with arrowheads indicating symphalangism in the hands of patient #991. (**b**) Audiograms from patient #991 showing conductive hearing loss.

As shown in the pedigree (Fig. S1a), patient #991 was diagnosed with symmetric conductive hearing loss of 50 dB (Fig. 1b) at the age of 6 years. Tympanography indicated type A sclerosis and absence of the stapedius reflex, whereas otomicroscopy results were normal. Temporal bone computed tomography (CT) scan revealed no inner or middle ear malformations. Her hearing level was stable and non-progressive, and she received hearing aids in both ears. At the age of 17, exploratory tympanotomy of the left ear showed bony fixation of the footplate without any other deformities in the middle ear and stapedotomy using a Teflon piston and wire was performed, resulting in a remarkable improvement in hearing. One year later, stapedotomy was undertaken in her right ear also. After the surgery, the postoperative hearing levels showed 20–30 dB and she did not use her hearing aids. The X-ray presented in Fig. 1 shows symphalangism in the PIP joints of the second to fifth fingers of both hands and in the distal interphalangeal (DIP) joints of the left second and fifth fingers and of the right fifth finger. There was symphalangism in both hands, resulting in limited mobility of the fingers. Symphalangism (fixation of the proximal interphalangeal joint) in both feet was also found. The ankylosis was confirmed by X-ray examination (Fig. 1a). Congenital hyperopia (only in this patient within the family) was also present.

### Family 2

**Figure 2:**
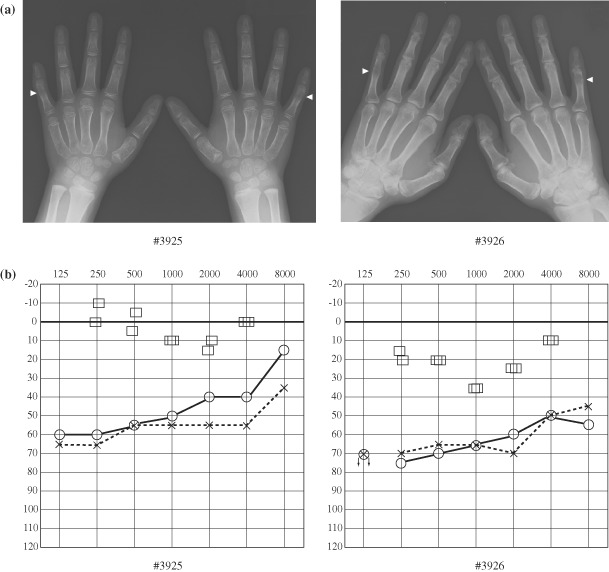
(**a**) Photograph with arrowheads indicating symphalangism in the hands of patients #3925 and #3926. (**b**) Audiograms from patients #3925 and #3926 showing conductive hearing loss.

As shown in the pedigree (Fig. S2a), a girl and her father (patients #3925 and #3926) visited our hospital due to bilateral hearing loss. Audiograms indicated bilateral mixed hearing loss (Fig. 2b). Anamnestic evaluation suggested that the hearing loss was non-progressive without any associated symptoms such as ear fullness, tinnitus or vertigo. Patient #3926 underwent stapedotomy at the age of 42, achieving significant recovery of his hearing. There was symphalangism in the PIP joint of both fifth fingers, resulting in limited mobility of the fingers. Fixation of the proximal interphalangeal joint was not found in either foot. The ankylosis was confirmed by X-ray examination (Fig. 2a). Congenital hyperopia was not present in this family.

### Family 3

**Figure 3:**
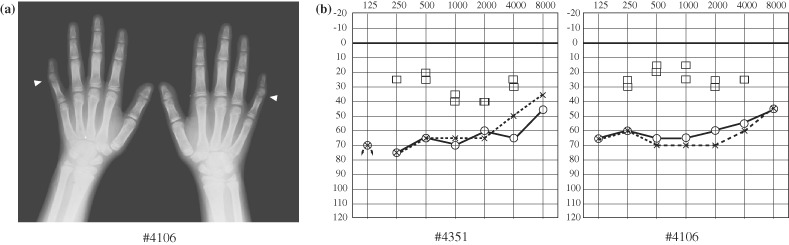
(**a**) Photograph with arrowheads indicating symphalangism in the hands of patients #4106. (**b**) Audiograms from patients #4106 and #4351 showing conductive hearing loss.

The pedigree shows hearing loss was inherited in four generations, indicating autosomal dominant inheritance (Fig. S3a). In addition to conductive hearing loss (Fig. 3b), the family members were associated with the following clinical phenotype in various degrees: hyperopia, broad thumbs and first toes, symphalangism, syndactyly, and fused cervical vertebrae (Fig. S3a). The clinical diagnosis was therefore stapes ankylosis with broad thumb and toes (3) or Teunissen and Cremer syndrome (7). Patient #4351 had conductive hearing loss, hyperopia, broad thumbs and first toes, symphalangism, and syndactyly. She noted her hearing loss around age 10. Stapedotomy was performed when she was 37 (right) and 38 (left) years old, achieving significant recovery of hearing. Patient #4106 had conductive hearing loss, hyperopia, broad thumbs and first toes, and fused cervical vertebrae. His hearing loss was noted around age 3 and was diagnosed at the age of 8. Stapedotomy was performed when he was 9 (right) and 10 (left) years old, achieving significant recovery of hearing.

### Mutation identification

Human *NOG* gene coding is constituted of one single exon, in which an open reading frame of 696 nucleotides encodes a NOG polypeptide of 232 amino acids. A sequence obtained from GeneBank U31202 was used to design primers containing the entire coding region of *NOG*. Two fragments to entirely cover the coding region of NOG were amplified with polymerase chain reaction (PCR) and two specific primer pairs, as follows: F1, 5′-CTTGTGTGCCTTTCTTCCGC-3′; R1, 5′-TACTGGATGGGAATCCAGCC-3′; and F2, 5′-TACGACCCAGGCTTCATGGC-3′; R2, 5′-TAGCACGAGCACTTGCACTC-3′.

PCR reactions were carried out in 25 µl total volume containing 40 ng of genomic DNA, 10 pmol of each primer, 2 mM dNTPs, ×10 PCR buffer and 0.2 U of ExTaq polymerase (Takara, Tokyo, Japan). PCR conditions were denaturing at 94°C for 2 min; 35 cycles at 96°C for 30 s, 60°C for 30 s, 72°C for 1 min extension, with a final extension step at 72°C for 5 min in a Perkin-Elmer 9600 thermal cycler (Perkin-Elmer, Foster City, CA). PCR products were purified with a Suprec filter (Takara) and sequenced directly, using four primers (F1, R1, F2, and R2) and ABI BigDye terminators, on an ABI 3100 sequencer (Perkin-Elmer).

## Results

Three novel mutations of the *NOG* gene were found by direct sequencing analysis in three families, whose common clinical features were compatible with SYM1, i.e. immobility of the PIP joints of the hands and toes, and congenital conductive hearing loss due to stapes ankylosis. Patient #991 of family 1 had a heterozygous G>T transversion at nucleotide 551 (Fig. S1b), predicting a cysteine (C) for phenylalanine (F) substitution at amino acid 184 (C184F) in the coding region of NOG. Since the C184F mutation was not found in either parent and was found only in the proband (patient #991), it was suggested that the mutation arose *de novo* in only the affected individual. Patients #3925 and #3926 of family 2 had a heterozygous T>A transversion at nucleotide 463 (Fig. S2b), predicting a cysteine (C) for serine (S) substitution at amino acid 155 (C155F) in the coding region of NOG. Patients #4106 and #4351 had a heterozygous C215X mutation.

None of the otosclerosis patients had *NOG* mutations. These three mutations were not observed in any of the other family members nor in the 192 unrelated Japanese controls (384 chromosomes).

## Discussion

This study identified three novel mutations in the *NOG* gene in families with symphalangism, being consistent with the previous work showing that *NOG* is the responsible gene for SYM1 and stapes ankylosis with broad thumb and toes/Teunissen and Cremer syndrome. One mutation was a nonsense mutation (C215X), leading to a truncated protein, and was likely to be a pathologic mutation. The other two mutations are also likely to be pathologic rather than functionally neutral polymorphic changes because: (i) none were found in any of the controls, (ii) the alignment of NOG sequences from human, mouse, chicken, *Xenopus laevis* and zebrafish showed that C155 and C184 are well-conserved amino acids in all species (data not shown), and (iii) all affected subjects showed similar phenotypes.

To date, 36 *NOG* mutations have been reported in SYM1, SYNS1, TCC, BDB2 and TCS families (Table 2). Although the *NOG* mutations have been reported mainly in dominant families (Table 2), *de novo NOG* mutations have also been reported in sporadic SYM1 (8) and sporadic SYNS1 (1). Therefore, genetic investigation may be needed for determining pathogenesis of congenital stapes ankylosis with stiffness of the PIP joints, even in sporadic cases. A milder phenotype (3) as well as the present case with minor joint anomalies in family 2 indicated that it may be clinically important to check such skeletal abnormalities when diagnosing and treating patients with stapes ankylosis, because it may be difficult to differentiate congenital stapes ankylosis from otosclerosis when conductive hearing loss is delayed to adulthood.

**Table 2:** NOG mutations reported in SYM1, SYNS1, TCC, BDB2 and TCS families

Nucleotide change	Amino acid	Family information	Phenotype	Evolutionary conservation +	Domain/structure/motif ++	Authors
c. 58delC	Frameshift	Japanese, AD	SYNS1	—	—	Takahashi (8)
c. 103C>G	p. P35A	German, AD	BDB2	Conserved	Finger/clip region Interface of NOG and BMP7	Lehmann (4)
c. 103C>T	p. P35S	Turkish, AD	BDB2	Conserved	Finger/clip region Interface of NOG and BMP7	Lehmann (4)
c. 103C>T	p. P35S	Israeli, AD	SABTT	Conserved	Finger/clip region Interface of NOG and BMP7	Hirshoren (9)
c. 103C>T	p. P35S	Italian, AD	SYM1	Conserved	Finger/clip region Interface of NOG and BMP7	Mangino (10)
c. 104C>G	p. P35R	NI, sporadic	SYM1	Conserved	Finger/clip region Interface of NOG and BMP7	Gong (1)
c. 104C>G	p. P35R	NI, AD	TCC	Conserved	Finger/clip region Interface of NOG and BMP7	Dixon (2)
c. 106G>C	p. A36P	Danish, AD	BDB2	Almost conserved^a^	Finger/clip region Interface of NOG and BMP7	Lehmann (4)
c. 110C>G	p. P37R	Belgian, AD	TCC	Conserved	Finger/clip region Interface of NOG and BMP7	Debeer (11)
c. 124C>G	p. P42A	Belgian, *de novo*	TCC	Conserved	Finger/clip region Interface of NOG and BMP7	Debeer (12)
c. 125C>G	p. P42R	NI, AD	SYNS1	Conserved	Finger/clip region Interface of NOG and BMP7	Oxley (13)
c. 129-130dup	Frameshift	Dutch, AD	SABTT	—	—	Weekamp (14)
c. 142G>A	p. E48K	Japanese, sporadic	SYM1	Conserved	Finger/clip region Interface of NOG and BMP7	Kosaki (15)
c. 142G>A	p. E48K	Iranian, AD	BDB2	Conserved	Finger/clip region Interface of NOG and BMP7	Lehmann (4)
c. 149C>G	p. P50R	Belgian, *de novo*	TCC	Conserved	Finger/clip region Interface of NOG and BMP7	Debeer (12)
c. 252-253 insC	Frameshift	NI, AD	SABTT	—	—	Brown (3)
c. 304delG	Frameshift	Dutch	SYM1	—	—	Thomeer (16)
c. 328C>T	p. Q110X	Italian, AD	SABTT	Conserved	—	Brown (3)
c. 386T>A	p. L129X	Japanese, AD	SYM1	Almost conserved^b^	—	Takahashi (8)
c. C391C>T	p. Q131X	Dutch	SYM1	Almost conserved^b^	—	Thomeer (16)
c. 463T>A	p. C155S	Japanese, AD	SYM1	Conserved	Conserved cysteine of cysteine knot I	Present study
c. 499C>G	p. R167G	North American, sporadic	BDB2	Conserved	—	Lehmann (4)
c. 551G>A	p. C184Y	Japanese, sporadic	SYM1	Conserved	Conserved cysteine of cysteine knot III	Takahashi (8)
c. 551G>T	p. C184F	Japanese, sporadic	SYM1	Conserved	Conserved cysteine of cysteine knot III	Present study
c. 559C>T	p. P187S	British, AD	BDB2	Conserved	—	Lehmann (4)
c. 561del	Frameshift	Dutch, AD	SABTT	—	—	Weekamp (14)
c. 565G>T	p. G189C	Dutch, AD	SYM1	Conserved	—	Gong (1)
c. 568A>G	p. M190V	NI, AD	SYNS1	Conserved	—	Oxley (13)
c. 608T>C	p. L203P	Dutch, AD	SABTT	Conserved	*β*-Sheet 3 of NOG structure	Weekamp (14)
c. 611G>T	p. R204L	NI, AD	TCC	Conserved	*β*-Sheet 3 of NOG structure	Dixon (2)
c. 614G>A	p. W205X	sporadic	SYNS1	Conserved	—	Dawson (17)
c. 615G>C	p. W205C	Belgian, AD	SYNS1	Conserved	*β*-Sheet 4 of NOG structure	Declau (18)
c. 615G>C	p. W205C	American, sporadic	SABTT	Conserved	*β*-Sheet 4 of NOG structure	Emery (19)
c. 645C>A	p. C215X	Japanese, AD	SABTT	Conserved	Disulphide bounds in cysteine knot motif to stabilize finger 2 structure	Present study
c. 649T>G	p. W217G	Hawaiian, AD	SYNS1	Conserved	*β* sheet 4 of NOG structure	Gong (1)
c. 659-660TC>AT	p. I220N	Belgian, AD	SYM1	Almost conserved^a^	Interaction region to BMP-type binding epitope	Gong (1)
c. 659T>A	p. I220N	NI, AD	SYM1	Almost conserved^a^	Interaction region to BMP-type binding epitope	Gong (1)
c. 664T>G	p. Y222D	Belgian, AD	SYM1	Conserved	Interaction region to BMP-type binding epitope	Gong (1)
c. 665A>G	p. Y222C	American, AD	SYM1	Conserved	Interaction region to BMP-type binding epitope	Gong (1)
c. 665A>G	p. Y222C	NI, AD	TCC	Conserved	Interaction region to BMP-type binding epitope	Dixon (2)
c. 668C>T	p. P223L	NI, AD	SYM1	Conserved	Interaction region to BMP-type binding epitope	Gong (1)
c. 696C>G	p. C232W	Germany, AD	SYM1	Conserved	Intermolecular disulphide bounds to stabilize NOG dimmer structure	Rudnik-Schöneborn (20)
17q22 long deletion		Japanese, sporadic	SYNS1	—	—	Shimizu (21)

+, evolutionary conservation was evaluated by the NCBI data base; ++, the domain/structure/motif are based on a hypothesized protein structure; AD, autosomal dominant; BDB2, brachydactyly type B2; BMP, bone morphogenetic protein; FOP, fibrodysplasia ossificans progressiva; NI, no information; NOG, noggin; SABTT, stapes ankylosis with broad thumbs and toes; SYNS1, multiple synostosis syndrome; SYM1, proximal symphalangism; TCC, trasal–carpal coalition syndrome.

^a^Residue is conserved across mammalians except for zebrafish.

^b^Residue is conserved across mammalians except for zebrafish and chicken.

Whether *NOG* mutations can be found more frequently in sporadic conductive hearing loss patients is an interesting question. In this study, mutations were not found in any otosclerosis patients who did not have any associated abnormality. Therefore, typical otosclerosis is not a continuum of the category of diseases associated with *NOG* mutations. These results, together with the previous literature, indicate that the *NOG* mutations are restrictively found within patients with various skeletal abnormalities regardless of severity. It is noted that the reported *NOG* mutation in mild cases (patients with stapes ankylosis without symphalangism) have minor skeletal abnormalities such as broad thumbs and great toes (3), but these cases had symphalangism in the little fingers only.

A review of the reported 41 mutations showed that, interestingly, the majority of *NOG* mutations are located in the evolutionally well conserved and therefore functionally critical region (Table 2), suggesting that this region might be functionally relevant in NOG polypeptides. This study added three novel *NOG* mutations in conserved cysteine residue within the cystine knot motif and confirmed that *NOG* is a causative gene for this category of disease. In addition, there was no particular racial-specific founder mutation within this gene (Table 2). With regard to a genotype–phenotype correlation, phenotypes seem to be independent of the location of the mutation and type of mutations (Table 2). Other genetic factors and/or interacted proteins may also be involved in determining clinical phenotypes.

With regard to surgical outcome, stapes surgery for conductive hearing loss due to *NOG* mutations may be a good therapeutic option for most cases. In fact, two of these seven patients who underwent stapes surgery (#991 and #4106) had hearing deterioration 3–10 years after the initial surgery, in accordance with a previous report (3, 22), hypothesizing that excessive bony overgrowth and refixation of the ossicle chain may occur after initially successful surgery. The other cases in this study maintained good hearing even after long-term follow-up periods (more than 10 years). Therefore, surgical outcome should be carefully evaluated after long-term observation. Careful explanation of possible limitations of surgical treatment and alternative treatment options such as a bone-anchored hearing aid may be appropriate for such patients with this genetic background.

The identification of the causative genes responsible for various middle/inner ear diseases will enable us to classify new congenital deafness groups in the future, and lead to clinical application in the diagnosis of middle ear disorders and better counseling for the selection of ideal intervention.

## Conflict of interest

The authors declare no conflict of interest.

## Supporting Information

Fig. S1. (**a**) Pedigree of family 1. Filled symbolrepresents the affected individual. (**b**) Sequence analysis ofnoggin (NOG). Arrow indicates a G to T change at nucleotide 551 inpatient &num;991. This substitution causes codon 184 to changefrom TGC (cysteine: C) to TTC (phenylalanine: F).

Fig. S2. (**a**) Pedigree of family 2. Filled symbolsrepresent the affected individuals. (**b**) Sequence analysis ofnoggin (NOG). Arrow indicates a T to A change at nucleotide 463 inpatients &num;3925 and &num;3926. This substitution causescodon 155 to change from TGC (cysteine: C) to AGC (serine: S).

Fig. S3. (**a**) Pedigree of family 3. Filled symbolsrepresent affected symptoms (conductive hearing impairment,hyperopia, and finger malformation. (**b**) Sequence analysis ofnoggin (NOG). Arrow indicates a C to A change at nucleotide 645 inpatients &num;4106 and &num;4351. These two patients had aheterozygous C215X mutation. This nonsense mutation (C215X) leadsto a truncated protein.
