# Supplementary material for: Mutations in the NOG gene are commonly found in congenital stapes ankylosis with symphalangism, but not in otosclerosis
Source: Clin Genet. 2012 Jan 30;82(6):514–20. doi: 10.1111/j.1399-0004.2011.01831.x (PMC3532604; doi:10.1111/j.1399-0004.2011.01831.x)

Supplementary Figure 1

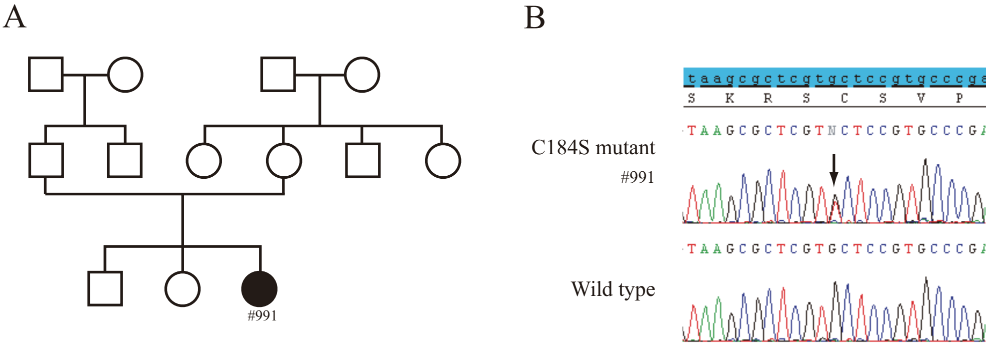

Supplement: Fig. S1. — (a) Pedigree of family 1. Filled symbolrepresents the affected individual. (b) Sequence analysis ofnoggin (NOG). Arrow indicates a G to T change at nucleotide 551 inpatient #991. This substitution causes codon 184 to changefrom TGC (cysteine: C) to TTC (phenylalanine: F). [file cge0082-0514-SD1.pdf]
