# Supplementary figures and images for: Mutations in the NOG gene are commonly found in congenital stapes ankylosis with symphalangism, but not in otosclerosis
Source: Clin Genet. 2012 Jan 30;82(6):514–20. doi: 10.1111/j.1399-0004.2011.01831.x (PMC3532604; doi:10.1111/j.1399-0004.2011.01831.x)

A

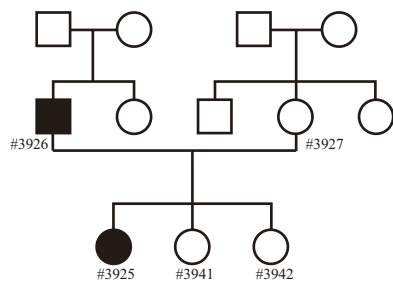

B

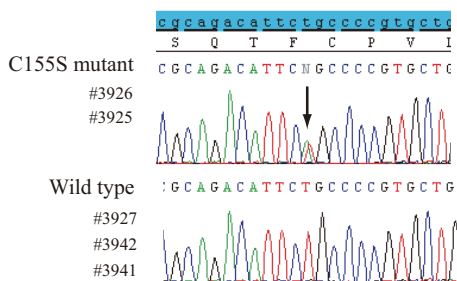

Supplement: Fig. S2. — (a) Pedigree of family 2. Filled symbolsrepresent the affected individuals. (b) Sequence analysis ofnoggin (NOG). Arrow indicates a T to A change at nucleotide 463 inpatients #3925 and #3926. This substitution causescodon 155 to change from TGC (cysteine: C) to AGC (serine: S). [file cge0082-0514-SD2.pdf]

A

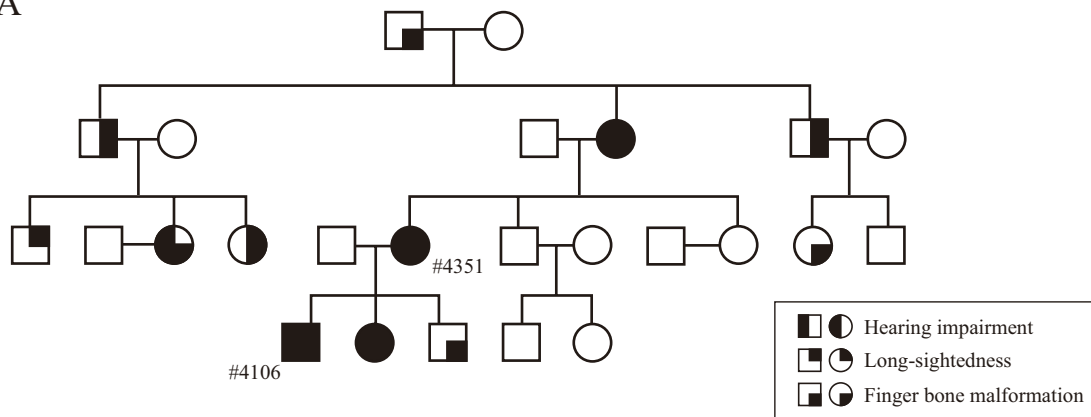

B

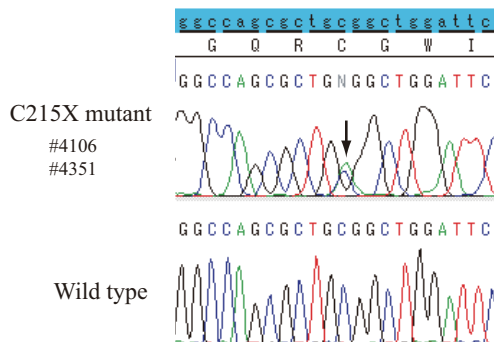

Supplement: Fig. S3. — (a) Pedigree of family 3. Filled symbolsrepresent affected symptoms (conductive hearing impairment,hyperopia, and finger malformation. (b) Sequence analysis ofnoggin (NOG). Arrow indicates a C to A change at nucleotide 645 inpatients #4106 and #4351. These two patients had aheterozygous C215X mutation. This nonsense mutation (C215X) leadsto a truncated protein. [file cge0082-0514-SD3.pdf]
